# 基于TM&M分级系统肺癌胸腔镜肺主要手术并发症列线图预测模型的建立和验证

**DOI:** 10.3779/j.issn.1009-3419.2021.103.12

**Published:** 2021-12-20

**Authors:** 轲 兰, 健 周, 海华 郭, 云峰 倪, 帆 杨

**Affiliations:** 1 100044 北京，北京大学人民医院胸外科 Department of Thoracic Surgery, Peking University People's Hospital, Beijing 100044, China; 2 710038 西安，空军军医大学唐都医院胸外科 Department of Thoracic Surgery, Tang Du Hospital, Air Force Military Medical University, Xi'an 710038, China

**Keywords:** 肺肿瘤, 电视辅助胸腔镜手术, 并发症, 预测模型, Lung neoplasms, Video assisted thoracoscopic surgery, Complication, Prediction model

## Abstract

**背景与目的:**

并发症是肺切除术后患者死亡的重要原因，目前我国肺癌胸腔镜手术普及率逐年增高，但肺癌胸腔镜手术术后并发症的预测模型尚缺乏基于大样本数据库的支持。本研究采用TM&M（Thoracic Mortality and Morbidity）分级系统全面描绘我中心胸腔镜肺癌手术术后并发症，并建立和验证并发症的预测模型。该模型可为此类患者术后并发症的预防和干预提供依据，从而加速患者康复。

**方法:**

回顾性收集我中心2007年1月-2018年12月胸腔镜肺癌手术患者临床资料，仅纳入Ⅰ期-Ⅲ期肺癌行胸腔镜肺主要手术的肺癌患者，术后并发症登记严格采用TM&M分级系统。将患者按照手术时期分为两组：前期组（2007年-2012年）和后期组（2013年-2018年），以倾向评分匹配法对两组基线数据进行匹配；匹配后数据采用二元*Logistic*回归分析建立并发症的预测模型，Bootstrap法内抽样进行内部验证。

**结果:**

研究共纳入2, 881例肺癌患者，平均年龄（61.0±10.1）岁，其中发生主要并发症180例（6.2%）。匹配后的1, 268例患者进行二元*Logistic*回归分析显示：年龄（OR=1.04, 95%CI: 1.02-1.06, *P* < 0.001）、手术时期（OR=0.62, 95%CI: 0.49-0.79, *P* < 0.001）、病理类型（OR=1.73, 95%CI: 1.24-2.41, *P*=0.001）、术中出血量（OR=1.001, 95%CI: 1.000-1.003, *P*=0.03）、清扫淋巴结数目（OR=1.022, 95%CI: 1.00-1.04, *P*=0.005）为术后并发症发生的独立危险因素；将其纳入列线图模型，受试者工作特征曲线（receiver operating characteristic curve, ROC）提示该模型区分度较好（C-指数为0.699），1, 000次重复Bootstrap内部抽样验证C-指数为0.680。校准曲线显示预测模型的校准度良好。

**结论:**

TM&M并发症分级系统可全面准确地报告胸腔镜肺癌外科的术后并发症。年龄、手术时期、病理类型、术中出血量、清扫淋巴结数目是胸腔镜肺癌手术后主要并发症的独立危险因素，以此建立的并发症预测模型具有较好的区分度和校准度。

## Abstract (en)

**Background and objective:**

Postoperative complications are an important cause of death after lung resection. At present, the adoption of video assisted thoracoscopic surgery (VATS) for lung cancer in China is increasing every year, but the prediction model of postoperative complications of VATS for lung cancer is still lack of evidence based on large sample database. In this study, Thoracic Mortality and Morbidity (TM&M) classification system was used to comprehensively describe the postoperative complications of VATS major lung resection in our center, and the prediction model of complications was established and verified. The model can provide basis for the prevention and intervention of postoperative complications in such patients, and accelerate the recovery of patients.

**Methods:**

The clinical data of patients underwent VATS major lung resection in our center from January 2007 to December 2018 were collected retrospectively. Only patients with stage Ⅰ-Ⅲ lung cancer were included. The postoperative complications were registered strictly by TM&M classification system. The patients were divided into two groups according to the operation period: the early phase group (From 2007 to 2012) and the late phase group (From 2013 to 2018). The baseline data of the two groups were matched by propensity score matching. After matching, binary *logistic* regression analysis was used to establish the prediction model of complications, and bootstrap internal sampling was used for internal verification.

**Results:**

A total of 2, 881 patients with lung cancer were included in the study, with an average age of (61.0±10.1) years, including 180 major complications (6.2%). Binary *Logistic* regression analysis of 1, 268 matched patients showed: age (OR=1.04, 95%CI: 1.02-1.06, *P* < 0.001), other period (OR=0.62, 95%CI: 0.49-0.79, *P* < 0.001), pathological type (OR=1.73, 95%CI: 1.24-2.41, *P*=0.001), blood loss (OR=1.001, 95%CI: 1.000-1.003, *P*=0.03), dissected lymph nodes (OR=1.022, 95%CI: 1.00-1.04, *P*=0.005) were independent risk factors for postoperative complications. The ROC curve indicates that the model has good discrimination (C-index=0.699), and the C-index is 0.680 verified by bootstrap internal sampling for 1, 000 times. The calibration curve shows a good calibration of the prediction model.

**Conclusion:**

TM&M system can comprehensively and accurately report the postoperative complications of thoracoscopic lung cancer surgery. Age, operative period, pathological type, intraoperative bleeding and dissected lymph nodes were independent risk factors for postoperative complications of VATS major lung resection for lung cancer. The established complication prediction model has good discrimination and calibration.

手术是肺癌综合治疗的重要手段，早期肺癌经根治性切除后5年生存率可达80%以上^[1, 2]^。手术在发挥治疗性作用的同时，也增加了患者的并发症风险。肺癌术后并发症是患者围术期死亡的主要原因，约占死亡原因的84%，也是延长住院时间、增加二次住院率及住院费用的重要因素^[3, 4]^。

因而，预防术后并发症成为肺癌外科的重要问题之一。旨在有效降低术后并发症的策略如：合理的术前心肺功能锻炼、优化的手术方式、标准化的护理监测等，近年得到较为广泛的认可和推广^[5-7]^。在并发症的管理中，最有效的措施就是预防，通过术前检查筛查高危人群，从而采取有效的预防措施降低并发症风险。而建立预测模型即是筛查及评估高危人群最为直接的工具。基于大样本数据的胸外科并发症模型主要来自于国外的多中心胸外科手术数据库。如基于美国胸外科医师协会（The Society of Thoracic Surgeons, STS）数据库的27, 844例^[8]^和欧洲胸外科医师协会（European Society of Thoracic Surgeons, ESTS）数据库47, 960例^[9]^肺切除手术的并发症预测模型。但由于各研究并发症采用的定义标准不一，降低了不同医疗机构之间数据、结论的可比性以及分析结果的一致性和推广性。

2008年由加拿大渥太华医院胸外科制定的TM&M（Thoracic Mortality and Morbidity Classification）分级系统^[10]^，将Claviden-Dindo并发症分级系统^[11]^与常见不良反应事件评价标准（Common Terminology Criteria for Adverse Events, CTCAE）4.0^[12]^结合，制定了专门用来评价胸外科手术后并发症的分级系统，对每个并发症进行了定义和分级。TM&M分级系统被多项研究^[13, 14]^采用，显示出良好的分级分类能力，增强了不同医疗机构术后并发症研究的可比性。目前国内研究缺乏采用该系统对我国胸腔镜手术的并发症进行长跨度的全面回顾。基于此，本研究目的在于采用TM&M分级系统全面描绘胸腔镜肺癌手术术后并发症发生情况，研究并发症危险因素并建立并发症预测模型。

## 1 资料与方法

### 1.1 患者资料

系统性回顾2007年1月-2018年12月在北京大学人民医院胸外科住院接受胸腔镜肺主要手术的肺癌患者，肺主要手术包括：肺叶切除术，复合肺叶切除术，支气管袖式切除术及全肺切除术。本研究入选标准：①年龄≥18岁；②病理分期为Ⅰ期-Ⅲ期[第八版肿瘤原发灶-淋巴结-转移（tumor-node-metastasis, TNM）分期]肺癌；③住院期间行电视辅助胸腔镜手术（video-assisted thoracic surgery, VATS）肺叶、复合肺叶、袖式及全肺+系统性淋巴结清扫，术后病理确认切缘为R0切除；④病历资料完整。排除标准：①术中探查无法根治性切除转为楔形切除者；②中转开胸患者；③Ⅳ期手术患者；④既往接受同侧胸腔手术的患者。收集患者的一般临床资料、手术相关数据和病理等基线数据。采用TM&M并发症分级系统总结其术后并发症发生情况及其等级，描绘全身各系统不同种类并发症发生率及其等级，其中Ⅰ级需要药物干预，Ⅱ级需药物或简单操作干预，两者为次要并发症；Ⅲ级需要手术干预，Ⅳ级需生命支持，两者为主要并发症，Ⅴ级为死亡。其中TM&M对肺泡胸膜瘘定义为因脏层胸膜的撕裂或不连续导致的肺漏气，且持续时间 > 5 d。支气管胸膜瘘定义为是支气管与胸膜间形成的异常通道。本研究纳入经支气管镜或手术明确的支气管胸膜瘘。气胸定义为气体进入胸膜腔造成的积气状态。其中Ⅰ级不需要干预处理，Ⅱ级需 > 5 d的胸腔闭式引流。

### 1.2 手术方法

本研究手术方式采用全胸腔镜肺叶切除术^[15]^进行肺癌根治术+系统性淋巴结清扫术。

### 1.3 预测模型建立的依据

预测模型的建立通过纳入既往报道的危险因素或有临床可能性的变量来构建多因素回归分析。建立预测模型需要满足以下3个条件：充足的样本量、多因素回归分析和模型的验证^[16]^。

进行多因素回归分析时，结局变量的样本量至少应为纳入变量的10倍，如纳入20个变量建立模型，则需要胸外科手术术后并发症至少200例，一般报道的胸外科手术并发症发生率在20%左右，需要的样本量至少为1, 000例。本研究经PSM后纳入1, 268例患者，符合预测模型建立的基本条件。

### 1.4 统计学分析

对于分类变量，采用频率和构成比进行统计描述；对于数值变量，若服从正态分布，采用均数士标准差表示，若不服从正态分布，则使用中位数描述其集中趋势，四分位数间距（Inter-Quartile Range, IQR）描述其离散趋势。为增强我中心前后两个时期（2007年-2012年和2013年-2018年）入院患者基线资料的可比性，减少混杂因素，本研究采用倾向评分匹配（propensity score match, PSM）进行1:1匹配（卡钳值取0.1），匹配后数据再次进行χ^2^检验比较并发症发生率；匹配后数据使用二元*Logistic*回归建立并发症的多因素预测模型。采用R语言及rms程序包建立列线图并验证预测模型的区分度（Discrimination）和校准度（Calibration）。校准度可用校准曲线和*Hosmer-Lemeshow*拟合优度检验（good of fit test）来评价。利用列线图预测出每位研究对象的并发症发生概率，并从低到高排成一个队列，根据百分位数将队列分为n组，然后分别计算每组研究对象预测并发症发生概率和相应的实际并发症发生概率的均值，并将两者结合起来作图得到*n*个校准点，最后将*n*个校准点连接起来得到预测校准曲线。预测模型的区分度，常用C-指数来进行衡量，C-指数在0.5-1之间波动，其值越接近于1说明列线图的预测能力越准确。本研究采用Bootstrap法进行有放回的重复内抽样1, 000次进行内部验证来纠正模型的过度拟合（overfitting）。

采用SPSS 23.0软件、R语言（3.6.3版本）及Excel 2017软件进行统计分析，*P* < 0.05为差异有统计学意义。

## 2 结果

### 2.1 患者一般临床病理特征

研究最终纳入2, 881例患者，平均年龄（61.0±10.1）岁，75岁以上老年患者261例（9.1%）。其中男性1, 470例（51.0%），女性1, 411例（49.0%），有吸烟史者1, 046例（36.3%），患者一般临床病理特征见表 1。所有患者均顺利完成手术，围术期死亡患者8例（0.3%），其中术后急性心肌梗死1例，急性肺动脉栓塞4例，肺部感染2例，血胸1例。术后主要并发症发生率为6.2%，次要并发症发生率为15.4%，死亡率为0.3%。不同级别并发症发生情况为：Ⅰ级71例（2.5%），Ⅱ级372例（12.9%），Ⅲ级178例（6.2%），Ⅳ级2例（0.1%），Ⅴ级8例（0.3%）。最常见的并发症是肺泡胸膜瘘、心房颤动、皮下气肿以及其他肺部和胸膜并发症（包括气胸、胸腔积液、肺炎、乳糜胸等）。155例（5.4%）肺泡胸膜瘘（> 5 d），多为次要并发症。房颤139例（4.8%），其中Ⅱ级发生率为4.7%。皮下气肿105例（3.64%），Ⅲa级最常见（1.3%）。其他系统并发症发生率均不超过5%（图 1）。

**表 1:** 倾向评分匹配法匹配前及匹配后患者临床病理信息 Clinicopathological characteristics between two period before and after propensity-score matching

Variables	Before propensity-score matching				After propensity-score matching		
	Early phase (*n*=660)	Late phase (*n*=2, 221)	*P*		Early phase (*n*=634)	Late phase (*n*=634)	*P*
Gender							
Male	346 (52.4)	1, 124 (50.6)	0.412		327 (51.6)	348 (54.9)	0.237
Famale	314 (47.6)	1, 097 (49.4)			307 (48.4)	286 (45.1)	
Age (yr)	62.3±10.6	60.6±9.9	< 0.001		62.0±10.6	60.8±10.3	0.043
Smoking history							
None	400 (60.6)	1, 435 (64.6)	0.060		394 (62.1)	362 (57.1)	0.067
Yet	260 (39.4)	786 (35.4)			240 (37.9)	272 (42.9)	
BMI (kg/m^2^)	24.0±3.2	24.5±12.8	0.292		24.0±3.2	24.0±3.3	0.976
CCI							
0	497 (75.3)	1, 718 (77.4)	0.237		478 (75.4)	507 (80.0)	0.281
1	121 (18.3)	366 (16.5)			116 (18.3)	97 (15.3)	
2	29 (4.4)	114 (5.1)			28 (4.4)	22 (3.5)	
3	11 (1.7)	20 (0.9)			10 (1.6)	8 (1.3)	
4	1 (0.2)	2 (0.1)			1 (0.2)	0 (0.0)	
≥5	0 (0.0)	1 (0.0)			1 (0.2)	0 (0.0)	
Diameter of main lesion (cm)	2.6±1.4	2.4±1.5	0.016		2.6±1.4	2.5±1.6	0.441
FEV_1_ (L)	2.6±4.1	2.6±2.6	0.833		2.5±0.7	2.5±0.6	0.026
FEV_1_%	94.0±17.4	96.5±17.2	0.002		94.5±16.8	95.0±17.5	0.603
ASA							
Ⅰ	101 (15.3)	348 (15.7)	0.760		100 (15.8)	122 (19.2)	0.242
Ⅱ	523 (79.2)	1, 766 (79.5)			499 (78.7)	485 (76.5)	
Ⅲ	35 (5.3)	106 (4.8)			34 (5.4)	27 (4.3)	
Ⅳ	1 (0.2)	1 (0.0)			1 (0.2)	0 (0.0)	
Operation time (min)	189±55	160±49	< 0.001		186±53	185±55	0.86
Tumor location							
Right upper lobe	239 (36.2)	762 (34.3)	0.634		229 (36.1)	222 (35.0)	0.724
Right lower lobe	124 (18.8)	447 (20.1)			60 (9.5)	55 (8.7)	
Right middle lobe	61 (9.2)	210 (9.5)			119 (18.8)	112 (17.7)	
Left upper lobe	144 (21.8)	453 (20.4)			139 (21.9)	141 (22.2)	
Left lower lobe	92 (13.9)	349 (15.7)			87 (13.7)	104 (16.4)	
Procedure							
Lobectomy	635 (96.2)	2, 126 (95.7)	0.198		611 (96.4)	603 (95.1)	0.19
Bilobectomy	18 (2.7)	46 (2.1)			16 (2.5)	15 (2.4)	
Pneumonectomy	4 (0.6)	22 (1.0)			4 (0.6)	5 (0.8)	
Sleeve lobectomy	3 (0.5)	27 (1.2)			3 (0.5)	11 (1.7)	
Blood loss (mL)	100 (50-200)	30 (50-100)	< 0.001		100 (50-200)	50 (30-100)	< 0.001
Dissected nodal stations	6±2	6±1	0.016		6±2	7±2	< 0.001
Dissected lymph nodes	18±8	15±7	< 0.001		18±8	18±8	0.894
Histologic subtype							
Squamous cell	95 (14.4)	291 (13.1)	0.094		88 (13.9)	93 (14.7)	0.586
Adenocarcinoma	511 (77.4)	1, 800 (81.0)			496 (78.2)	479 (75.6)	
Small cell	11 (1.7)	33 (1.5)			11 (1.7)	16 (2.5)	
Others	43 (6.5)	97 (4.4)			39 (6.2)	46 (7.3)	
Pathological stage							
Ⅰ	445 (67.4)	1, 600 (72.0)	0.072		427 (67.4)	416 (65.6)	0.689
Ⅱ	100 (15.2)	291 (13.1)			95 (15.0)	106 (16.7)	
Ⅲ	115 (17.4)	330 (14.9)			112 (17.7)	112 (17.7)	
Values are mean±SD or median (IQR) or *n* (%) unless otherwise noted. ASA: American Society of Anesthesiologists; BMI: body mass index; CCI: charlson comorbidity index; DLCO: diffusing capacity of the lungs for carbon monoxide; FEV_1_: forced expiratory volume in one second; IQR: interquartile range; SD: standard deviation.							

**图 1:**
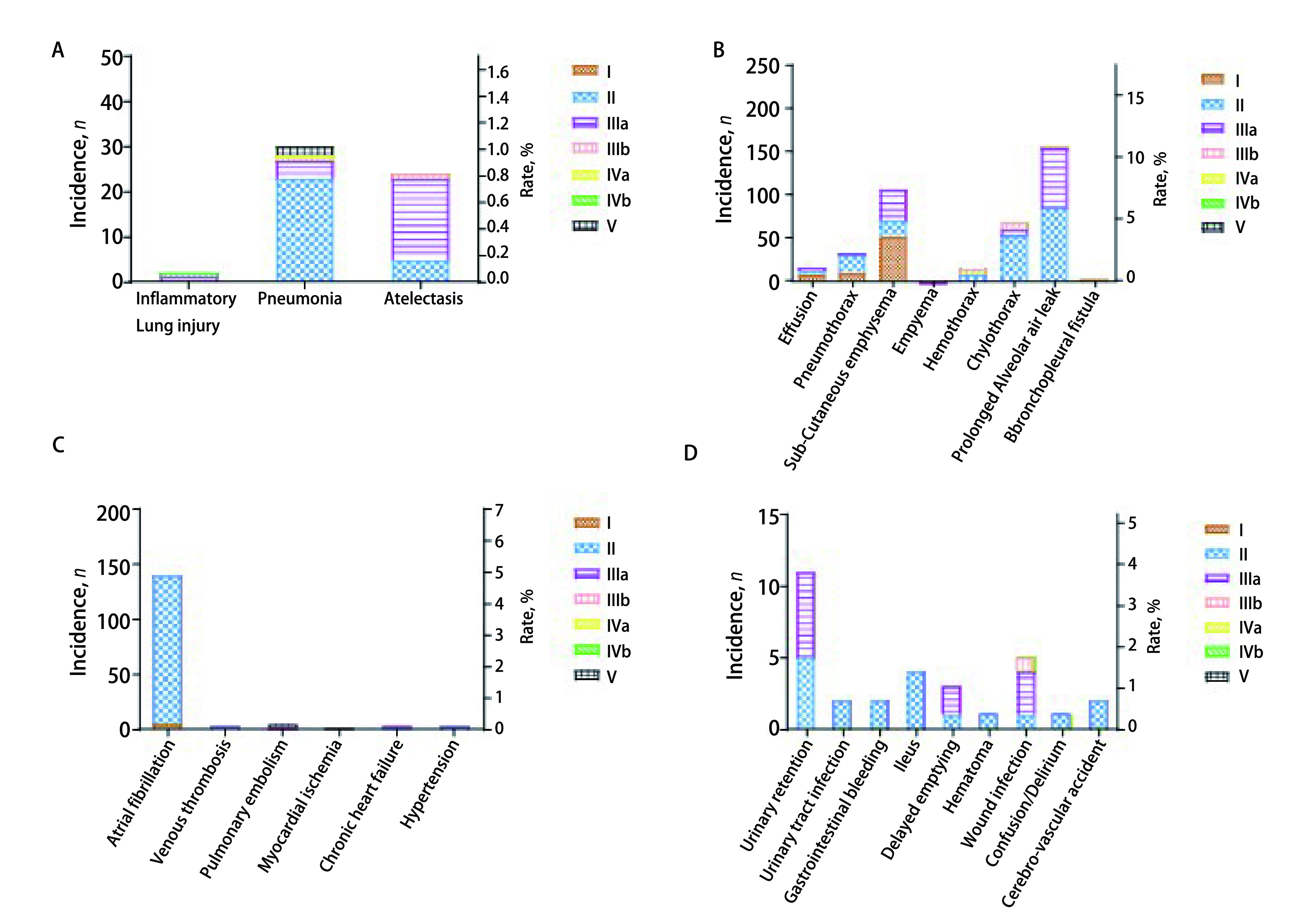
TM&M并发症发生率及等级。A：肺部并发症发生率及等级；B：胸膜腔并发症发生率及等级；C：心血管系统并发症发生率及等级；D：其他系统并发症发生率及等级。 The incidence rate and grade of complications classified by the TMM system. A: Pulmonary complications; B: Pleural complications; C: Cardiovascular system complications; D: Other system complications. TM&M: Thoracic Mortality and Morbidity.

### 2.2 倾向评分匹配结果

最终经PSM后有634对匹配成功，根据表3可以看出，在匹配前两组有8项变量有统计学差异，经匹配后降为4个，两组间数据平衡性较前明显提高。根据匹配后数据比较的前后期并发症存在差异（*P* < 0.001）。

### 2.3 匹配后数据建立并发症预测模型*P*（总并发症发生率）=e^x^/（1+e^x^）

其中x=-(-1.851-0.473^*^后期+1.166^*^其他病理类型+0.03^9*^年龄+0.001^*^出血量-0.125^*^清扫淋巴结站数+0.022^*^清扫淋巴结数目)

注：e为自然对数

将*Logistic*多因素回归分析得出的危险因素（表 2）对应的变量值纳入预测模型中，经R语言编程后制作出列线图。如图 2所示，该列线图包括年龄、病理类型、手术时期、出血量、清扫淋巴结站数、清扫淋巴结数6个最终指示变量。其中阶段1代表前期手术，0代表后期手术；病理类型1为鳞癌，2为腺癌，3为小细胞肺癌，4为其他病理类型。输入患者上述6个变量值，对照第一行的得分值，计算出总分值，总分值对应的并发症风险即为该患者预测的并发症概率。该模型受试者工作特征曲线（receiver operating characteristic curve, ROC）显示该模型的C-指数为0.699（95%CI: 0.670-0.727, *P* < 0.001），列线图总分为137时，并发症发生率为20.1%，此时约登指数（灵敏度+特异度-1）最大，模型的灵敏度为60.1%，特异度72.4%，此节点为并发症危险度的截断值（cut-off point）。

**表 2:** 并发症的二元*Logistic*回归分析 Binary *Logistic* regression analysis of complications

Variables	B	SE	Z	*P*	OR	95%CI	
Age	0.039	0.009	18.564	< 0.001	1.040	1.022	1.059
Late phase	-0.473	0.122	15.026	< 0.001	0.623	0.491	0.791
Other histologic subtype	1.166	0.503	5.383	0.02	3.211	1.198	8.601
Blood loss	0.001	0.001	0.058	0.031	1.001	1.000	1.002
Dissected nodal stations	-0.125	0.044	4.643	0.005	0.883	0.809	0.963
Dissected lymph nodes	0.022	0.009	5.689	0.017	1.022	1.004	1.040

**图 2:**
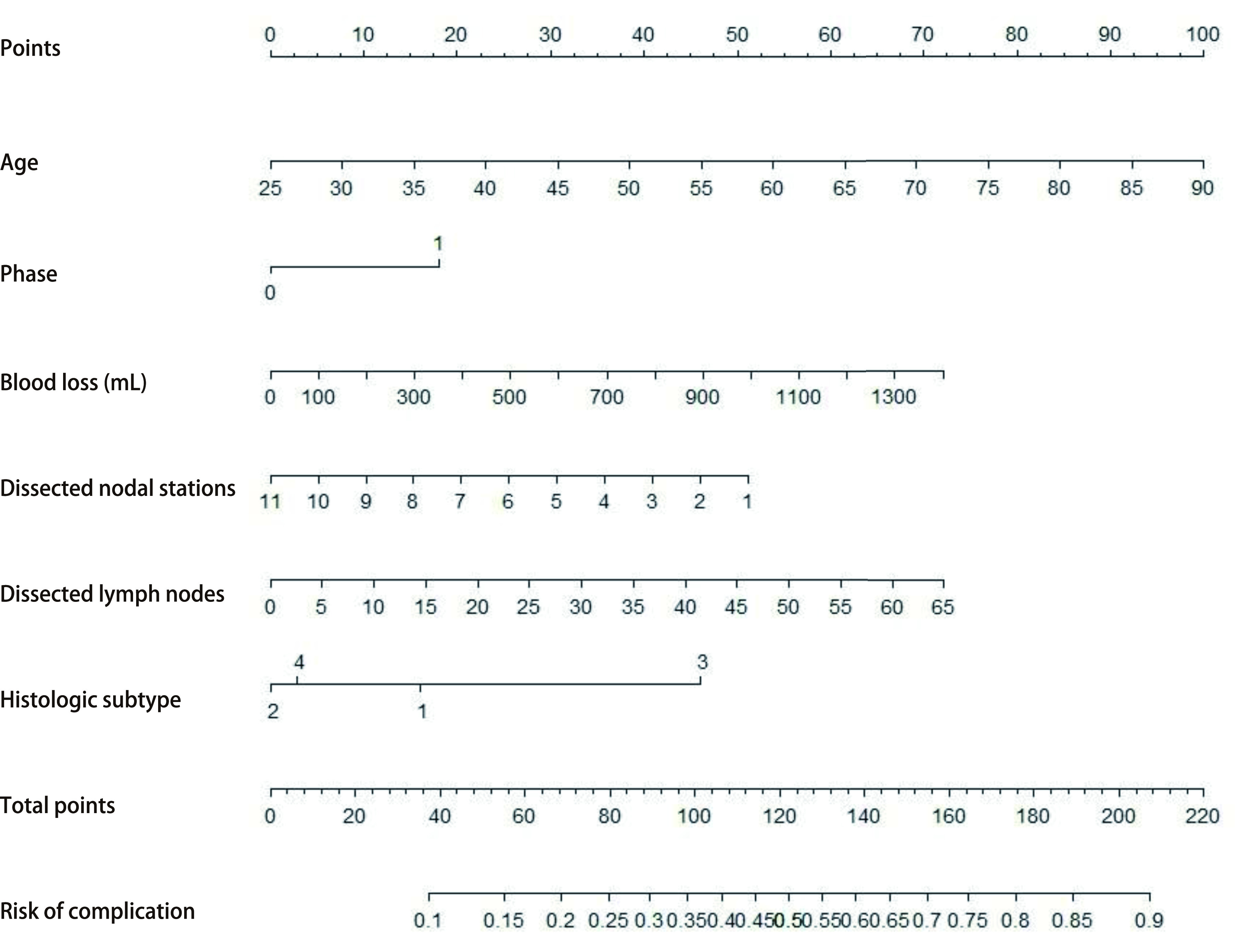
并发症风险预测列线图 Nomogram for risk prediction of complications

**图 3:**
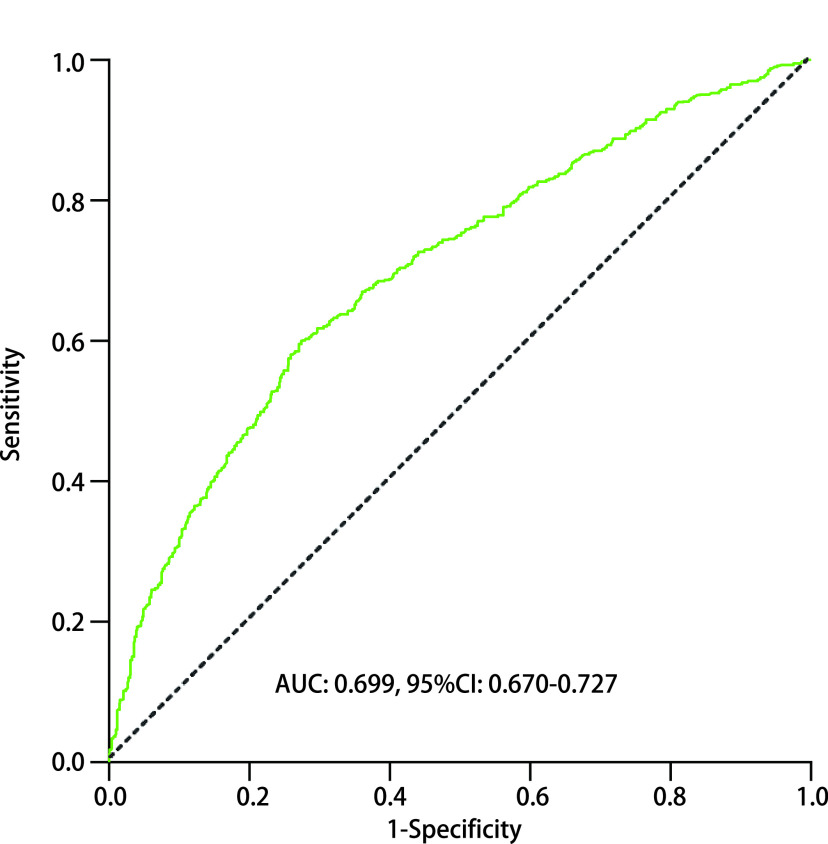
并发症预测模型ROC曲线（AUC：0.699） ROC curve of complication prediction model. AUC: 0.699; ROC: receiver operating characteristic curve; AUC: area under the curve.

### 2.4 预测模型的内部验证

经Bootstrap自抽样1, 000次对样本人群进行有放回的重复抽样，抽样样本经验证后，该模型预测并发症的C-指数为0.680，显示了较高的预测区分度（Discrimination），*Hosmer-Lemeshow*检验（*P*=0.755）显示该模型拟合度良好。

### 2.5 预测模型的校准度

图 4的校准曲线显示的预测值与实际值高度吻合，平均绝对误差为0.008，结合*Hosmer-Lemeshow*检验（*P*=0.755），综合反映该预测模型的校准度（Calibration）较高。

**图 4:**
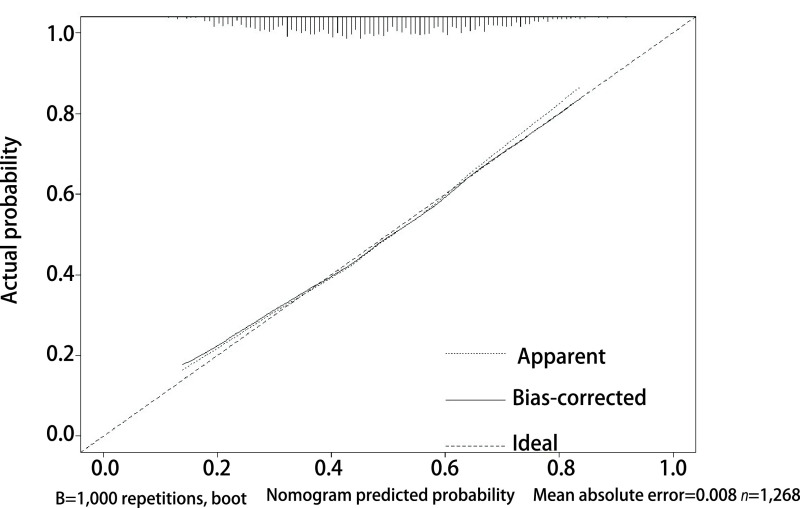
并发症预测模型的校准曲线（*n*=1, 268，平均绝对误差=0.008），X轴为预测模型的并发症概率，Y轴为实际的并发症概率。 Calibration curve of complication prediction model (*n*=1, 268, mean absolute error=0.008). The X-axis is the complication probability of the prediction model, and the Y-axis is the actual complication probability.

## 3 讨论

术前对患者进行有效的、客观的风险评估对临床医生和患者均有极大的获益。然而，由于并发症的复杂性和多样性使临床上评估工作的实施面临诸多困难。一个简单、合理、有效、重复性好的术后并发症评价系统有助于临床医生制定相应的诊疗策略，并降低不同医疗机构的差异引起的偏倚，最终指导患者临床结局的改善。

自2008年TM&M分级系统提出以来，已有多项研究证明了其在胸外科应用的可行性和可重复性。国内研究^[17, 18]^已有采用TM&M分级系统探讨胸外科并发症，证明其在我国并发症研究中的可行性。本研究采用TM&M分级系统全面分析我中心12年2, 881例患者的并发症发生情况，这种大样本、长跨度的针对肺癌胸腔镜肺主要手术的并发症分析，具有较好的样本代表性。本研究中，为得到相对一致的样本数据特性，我们排除了肺楔形、肺段及中转开胸手术的患者，避免了不同手术操作引起的偏移。同时，我们对患者的并发症进行了准确的分类分级，共分类了8个系统27种并发症发生情况。另外，本研究纳入的变量缺失值很少（0.46%），数据的完整性保证了统计分析的准确性。

本研究采用倾向评分匹配法平衡我中心前后期患者的基线数据，主要依据有：①胸腔镜手术技术及器械的不断进步^[19]^；②患者的临床特征不断变化，包括年龄、病理、分期、并发症、吸烟史等^[20]^，这些因素均为潜在的并发症危险因素。本研究利用匹配后的1, 268例患者数据，经二元*Logistic*回归分析发现，年龄、手术时期、病理类型、术中出血量、清扫淋巴结数目是胸腔镜肺癌肺主要手术后并发症的独立危险因素，这一结果与既往研究^[13, 21, 22]^类似。

随着人口老龄化的加剧及心脑血管疾病的高发，高龄肺癌患者往往伴有较多的合并症，术后并发症风险随之升高，然而，目前高龄并非肺癌手术的绝对禁忌，Okada等^[23]^的研究认为高龄（> 75岁）肺癌患者行肺叶或亚肺叶切除有良好的生存获益（5年生存率：肺叶为67.2%，亚肺叶为73.9%，*P*=0.94），术后主要并发症发生率分别为10.4%和5.1%，但两者间未见统计学差异（*P*=0.16）。本研究中年龄作为术后并发症的独立危险因素（*P* < 0.001），随着年龄的增加，术后并发症风险逐渐增加（OR=1.04, 95%CI: 1.02-1.06）。

鳞癌多为靠近段支气管以上的中央型肺癌，而腺癌多为周围型肺癌，小细胞肺癌则更易伴有纵隔淋巴结转移，因而一般认为小细胞肺癌、鳞癌的手术难度要大于腺癌；不同肺癌侵犯周围结构的倾向不同，组织粘连也不尽相同，故而不同病理类型可能会导致不同的并发症发生率。一项来自意大利的多中心前瞻性队列研究^[13]^报道了病理类型在术后并发症中的预测作用。通过纳入意大利国家数据库49个胸外科单位的4, 191例胸腔镜肺叶切除术的肺癌患者，经多因素回归分析，病理类型是术后并发症的危险因素之一（OR=1.73, 95%CI: 1.24-2.41, *P*=0.001）。本研究中病理类型作为术后并发症的危险因素，与上述报道类似。进一步对我中心不同病理类型的并发症进行亚组分析，发现腺癌（29.0%）、鳞癌（48.4%）、小细胞肺癌（52.3%）及其他病理类型肺癌（39.3%）术后并发症发生率差异有统计学意义（*P*=0.001），但这种统计学差异也可能与小细胞肺癌及其他病理类型的手术量较少有关，这需要进一步的大样本数据验证。

既往研究比较了不同手术经验的手术单位在并发症发生率上的不同，其中有研究^[13]^指出年手术量大于200例的中心是术后并发症的保护因素（OR=0.69, 95%CI: 0.55-0.89, *P* < 0.001）。我们的研究可以看作是自身前后对照，2007年-2011年我中心年胸腔镜肺癌肺主要手术量均不足200例，2012年-2018年则从212例逐渐升高到515例。经多因素分析，手术时期的后期是术后并发症的保护因素（OR=0.62, 95%CI: 0.49-0.79, *P* < 0.001），即手术时期的前期是术后并发症的危险因素。因此，对于开展胸腔镜肺癌根治术的单位，手术量的积累是保障手术质量的必要步骤。

既往研究^[8]^表明，纵隔淋巴结阳性是术后并发症的危险因素。本研究提出的清扫淋巴结数目则进一步细化了这一指标，而既往研究均未涉及这一变量，故该变量可能是胸腔镜肺癌肺主要手术术后并发症发生新的预测指标，ROC曲线也验证了淋巴结清扫数目对于术后并发症的预测能力。

列线图作为一种简单实用的个体化预测工具，在肿瘤生存预后的预测研究中得到广泛应用^[24, 25]^。本研究采用大样本单中心1, 268例肺癌患者基于TM&M系统建立预测模型，经内部验证和校准曲线显示较好的区分度和校准度，符合预测模型的基本条件，也是国内报道的大宗基于TM&M系统并发症的预测模型。本研究基于TM&M系统，建立的模型包含年龄、病理类型、出血量、手术时期、清扫淋巴结站数及清扫淋巴结数目等变量，模型的C-指数为0.699，相比于STS数据库主要并发症预测模型（C=0.68）^[8]^，ESTS数据库主要并发症预测模型（C=0.68）^[9]^，均略显优势，该模型对肺癌患者的术前准备和术后管理的临床决策提供了基于国人数据的重要参考工具。列线图在临床应用中方便实用，为医患双方的沟通提供了可视化的计算工具，提供危险因素计算的手术并发症概率，有利于增强患者对手术的理解和预防措施实施的配合。

本研究还有以下不足之处。由于研究是对单中心的数据库进行回顾性分析，这些结果可能会受到选择偏倚的影响，并限制结论的推广。因此本研究后续将进行多中心外部数据的验证。其次，由于我们的分析时间跨度较大，患者的围术期管理、相关的诊治手段均在不断发展，尤其是术后加速康复（enhanced recovery after surgery, ERAS）^[26]^及医疗系统改革的实践，因此并发症的预测具有一定的时限性，在不同时期、不同医疗机构间存在异质性。

综上所述，本研究基于TM&M分级系统分析我中心12年间2, 881例肺癌患者胸腔镜手术并发症发生情况并建立并发症预测模型，制作的列线图在临床上方便实用。未来，还需要更多区域中心的数据来增加该模型的外推性，从而构建基于国人大数据的并发症预测模型。
